# Parental Awareness, Knowledge, and Practices Regarding Speech and Language Delay in Children From the Eastern Region of Saudi Arabia: A Cross-Sectional Study

**DOI:** 10.7759/cureus.81248

**Published:** 2025-03-26

**Authors:** Mousa Alramadhan, Noura Almatroushi, Anwaar Mubarak, Jawad Alali, Sharifah Tumayhi, Aisha Alarfaj, Ghala Alsughayyir, Ahmad Al-Ali, Ali Alnajim, Hanadi Albatati

**Affiliations:** 1 Pediatrics and Child Health, King Faisal University, Hofuf, SAU; 2 Pediatrics and Child Health, National University of Science and Technology, Muscat, OMN; 3 Pediatrics and Child Health, Maternity and Children Hospital, Dammam, SAU; 4 Pharmacology, Al Laith General Hospital, Jeddah, SAU; 5 Pediatrics, King Saud Bin Abdulaziz University for Health Sciences, Riyadh, SAU; 6 Speech-Language Pathology and Audiology, King Abdulaziz University Faculty of Medicine, Jeddah, SAU

**Keywords:** child behaviour and development, parents attitude, parents’ knowledge, saudi arabian parents, speech language pathology

## Abstract

Background

The development of communication and language skills is a fundamental component of early childhood, serving as the foundation for initial learning experiences and the development of social skills. This study was designed to assess parental awareness, knowledge, and practices regarding speech and language delays in children.

Methodology

A cross-sectional survey was conducted among parents residing in the eastern region of Saudi Arabia. A total of 422 parents with at least one child under the age of 18 actively participated in the survey. A Google survey of 41 questions, including demographic information, knowledge, and practice sections, was randomly distributed nationwide from April to May 2024.

Results

The respondents were mostly Saudi nationals (n=388; 91.9%), and more than two-thirds of the participants were women (n=292; 69.19%). The study highlighted that 60.19% of the participants had poor knowledge of speech and language milestones, while only 1.18% demonstrated good knowledge. The primary sources of children's milestones include relatives and friends (62.9%) as well as the internet and social media (58.7%). Early intervention was recognized as beneficial, with 217 (51.42%) of parents acknowledging its potential to reduce treatment costs and durations, emphasizing the importance of timely detection and intervention programs.

Conclusions

This study shows that most parents had poor knowledge regarding language milestones and speech delay. Findings underscore the significance of addressing parental awareness and knowledge gaps concerning speech and language delays in Saudi Arabia, emphasizing the need for targeted educational campaigns. Improving awareness and correcting misconceptions can lead to more effective support systems for children with speech and language delays.

## Introduction

Speech can be described as the production of language in a verbal way, and language is defined as the conceptual processing of communication [1]. Language has two components: receptive language, which represents understanding the meaning of words and sentences, and expressive language, which represents the use of sounds, gestures, and the spoken word for communication [2]. Speech delay in children can be described as a delay in the use and development of the mechanisms necessary for speech production [3]. Language delay is when children acquire language more slowly compared to children in their age [4]. These problems can persist as a child grows up.

Speech and language delays are serious concerns because they are related to a child's overall development and impact both parents and the surroundings, particularly when the child reaches school age. School-aged children with speech or language delays may have learning and literacy problems, according to observational evidence [5]. The development of speech and language skills varies among children. They follow a natural timeframe for developing language skills. It can be detected as health professionals assess a child's progress and need for additional support by following a checklist of milestones [6]. Also, parental concerns, observations, and milestone assessment help identify speech and language abnormalities [7]. Several studies have reported the prevalence of speech and language delay in children, with different percentages. For example, a study conducted at the under-five clinic of JIPMER in Puducherry, India, reported that 27% of children under the age of three experienced speech and language delays [8].

A delay in speech and language development might be a symptom of many disorders, including mental retardation, hearing loss, an expressive language disorder, psychosocial deprivation, autism, selective mutism, receptive aphasia, and cerebral palsy. The physician needs to be familiar with the factors that affect speech when asking about history and physical examination in order to detect the delay as early as possible or to give a spot diagnosis. Early detection and intervention may allow the physician to identify emotional, social, and cognitive deficits associated with this condition, leading to better outcomes [3]. Parents’ awareness of their children's speech and language delay, affected by their perception of speech and language development, usually encourages them to seek professional services for assistance [9].

Speech and language delay in children is often associated with increased challenges in reading, writing, attention, and socialization [1]. Several studies from different countries demonstrated the prevalence of language delay among children. For example, a systematic review of the literature related to screening for speech and language delay in the UK stated that the estimated prevalence of speech and language delay in children 2-5 years of age is between 5% and 12%, with a median of 6% [10]. In India, a study published in 2019 found language delay in about 2.53% of children aged one to 12 years old [11]. Furthermore, in Iraq, the frequency of language delay was 11.9% among children under seven years of age [12]. Locally, in the Kingdom of Saudi Arabia and among preschool children, the overall prevalence of language delay was dramatically higher, reaching up to 24.5% according to a study conducted in 2017 [13].

Regarding parents’ knowledge, different studies were conducted to investigate their knowledge of developmental milestones in different developmental domains. For example, in Jeddah, Saudi Arabia, a cross-sectional study included 376 parents to assess their knowledge in four domains of children’s development. The motor domain had the highest level of accuracy, followed closely by the cognitive domain, while the language and social domains showed lower levels of accuracy [14]. Another study from Iraq assessing mothers' knowledge found that the highest scores were regarding children's language and hearing development milestones (59%) [15]. Most studies reported in the literature focus on assessing developmental milestones, with language being a component of that investigation. However, we also need to assess parents' awareness of speech and language delays in their children and how they respond based on their level of knowledge. Additionally, this study aims to identify gaps in parents' knowledge regarding potential causes of speech and language issues in children in Saudi Arabia.

## Materials and methods

Study methodology

This cross-sectional study was conducted to investigate parents’ awareness, knowledge, and practices regarding speech and language delay in children. This study was conducted between April to May 2024 using Google Forms. The survey was open to parents across all regions of Saudi Arabia with at least one child under 18, and they were invited to participate online. The study was approved by the Research Ethics Committee at King Faisal University (approval number: ETHICS2056). This study utilized convenience sampling techniques to collect participants. All information obtained from this research is used for scientific purposes alone, and participants’ data is obscured.

Inclusion and exclusion criteria

The study included parents living in Saudi Arabia who had at least one child under the age of 18, regardless of the child's gender. Participants must be residing in Saudi Arabia and can be of any nationality. The exclusion criteria were individuals without offspring or parents with offspring above 18 years of age. In addition, speech therapists were excluded from participation. The sampling technique used is convenient sampling where the questionnaire was distributed on social media platforms with a total of 422 participants.

Study procedure

Participants who provided their consent to participate in the study and met the inclusion criteria were accepted to be a part of the study. No personal information was requested from participants and each participant completed the questionnaire anonymously.

Data management

The questionnaire results were stored in a Microsoft Excel sheet (Microsoft Corp., Redmond, WA). Only individuals responsible for the statistical analysis had access to the results. No personal information was requested from participants that might threaten their confidentiality.

Questionnaire criteria

The questionnaire had 41 items. Starting first with the sociodemographic data, including nationality, residency, gender, age, and socioeconomic status. Followed by an assessment of parents' general knowledge regarding speech and language milestones, in addition to speech and language delay signs among children. The questionnaire also included multiple questions to know the sources of participants' knowledge about speech and language delay in children, an assessment of parental practice, and finally, an assessment of the association between knowledge categories and parental practice. The questionnaire was formulated based on a literature review to address the research's objectives. The content of the questionnaire was reviewed and verified by a specialist to ensure its pertinence, precision, and efficacy. The survey was first in English and then translated into Arabic to be understandable to the targeted populations. The Arabic version was approved after it was examined by a specialist.

Statistical analysis

This research was conducted to assess knowledge among parents, and a scoring system was utilized to categorize their knowledge levels as follows: Good knowledge (12-16), acceptable knowledge (7-11), and poor knowledge (0-6). There are 16 questions for assessing parents' knowledge regarding language milestones and speech-language delay as an overall knowledge assessment. The participants' choice of each question is presented as percentages. The minimum sample size was calculated using a single proportion sample size formula, assuming a 50% proportion, a 95% confidence level, and a 5% margin of error. This calculation indicated a minimum requirement of 385 participants. The mean and standard deviation (SD) of the overall knowledge scores were calculated and reported. The chi-square (x2) test was employed to analyze associations between knowledge categories and parental practice. Statistical significance was set at p-values <0.05. All statistical analyses were performed using JMP statistical software version 16 (SAS Institute Inc., Cary, NC).

## Results

Table 1 presents the socio-demographic data of all participants (n = 422), the majority of whom were Saudi (91.94%) and women (69.19%). Around 38.86% of respondents had four or more children, the majority of whom were predominantly breastfed (76.30%). Of the participants, 43.60% reported that their children have more than two hours of screen time. TV is the most used device (68.6%), followed by smartphones (43.4%) and tablets (31.4%).

**Table 1:** Sociodemographic Characteristics of the Study Population (N=422)

Variables	Values; n(%)
Nationality	
Saudi	388(91.94)
Non-Saudi	34(8.06)
Residency	
Northern Region	18(4.27)
Central Region	74(17.54)
Southern Region	22(5.21)
Eastern Region	278(65.88)
Western Region	30(7.11)
Gender	
Male	130(30.81)
Female	292(69.19)
Age range (in years)	
18-20	11(2.61)
21-30	112(26.54)
31-40	111(26.30)
41-50	114(27.01)
51-60	58(13.74)
61 and above	16(3.79)
Marital Status	
Married	387(91.71)
Divorced	18(4.27)
Widowed	17(4.03)
Highest level of education completed by the mother	
No formal education/illiterate	7(1.66)
School education	72(17.06)
Undergraduate degree	297(70.38)
Postgraduate degree	46(10.90)
Highest level of education completed by the father	
No formal education/illiterate	6(1.42)
School education	86(20.38)
Undergraduate degree	262(62.09)
Postgraduate degree	68(16.11)
Mother's current employment status	
Employed	185(43.84)
Housewife	193(45.73)
Retired	44(10.43)
Father’s current employment status	
Employed	338(80.09)
Unemployment	12(2.84)
Retired	72(17.06)
Household monthly income	
Less than 5000 SR	38(9)
5000 to 10,000 SR	102(24.17)
10,001 to 15,000 SR	104(24.64)
More than 15,000 SR	178(42.18)
Number of children	
One child	90(21.33)
Two children	88(20.85)
Three children	80(18.96)
Four or more children	164(38.86)
Do the children live with both parents?	
Yes	389(92.18)
No	33(7.82)
Have any of your children attended nursery?	
Yes	222(52.61)
No	200(47.39)
Have most of your children been predominantly breastfed?	
Yes	322(76.30)
No	100(23.70)
If yes, what was the average duration of breastfeeding?	
Less than 6 months	72(22.36)
6 to less than 12 months	123(38.20)
12 to 24 months	116(36.02)
More than 24 months	11(3.42)
How much screen time do your children have per day (including TV, computers, tablets, and phones)?	
No screen time	36(8.53)
Up to 1 hour	89(21.09)
Up to 2 hours	113(26.78)
More than 2 hours	184(43.60)
Type of device used most often by your children	
Tablet	117(31.4)
Smartphone	162(43.4)
TV	256(68.6)
Computer	33(8.8)
Other	14(4)

**Figure 1:**
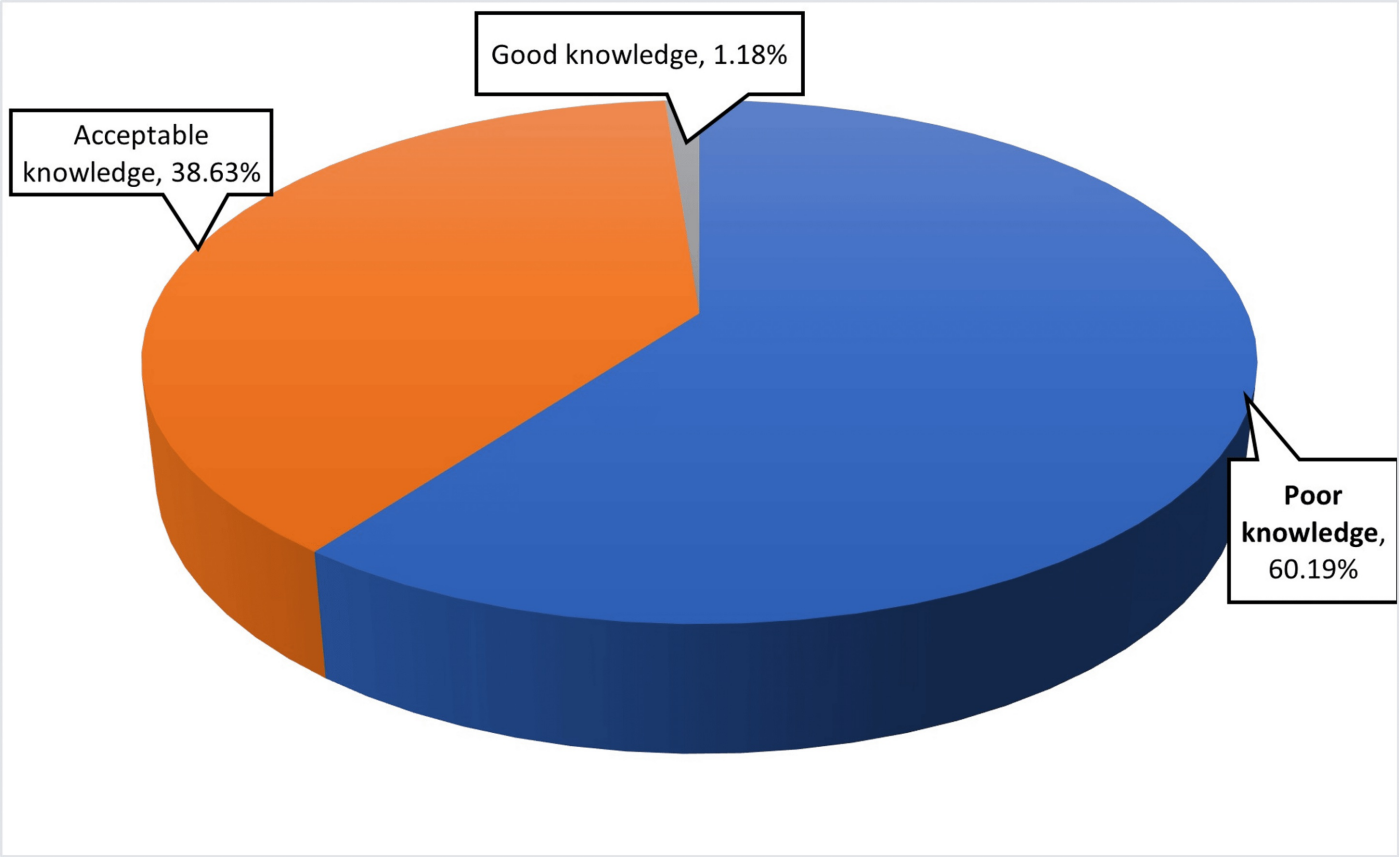
Summary of Overall Knowledge of Language Milestones and Speech-Language Delay

Table 2 represents findings regarding the knowledge and awareness of speech and language milestones and delay. The majority of participants (50.24%) correctly identified the capacity of a child to vocalize by four months, while a smaller portion (17.30%) thought this should occur by one month. When asked when a child generally says "Mama" or "Baba" nonspecifically, most people (60.19%) said nine months, with 25.59% believing it happens by 12 months. On the other hand, responses to calling the mother or father "Mama" and "Baba" have significant differences, with 34.60% selecting 12 months and 32.46% selecting 18 months. When asked at what age a child should be able to follow a one-step verbal command, 47.63% answered 12-15 months. Regarding building a vocabulary, 31.52% believed that a child should have a vocabulary of 50 or more words by the age of three, whereas 30.81% implied this milestone should be reached by 24-30 months. Most participants (91.71%) stated that they have heard about speech and language delays in children. However, detailed knowledge about language delay varied. In terms of knowledge, 48.32% categorized their knowledge as moderate, while 42.89% considered it as basic. Of the participants, 42.18% believed most children with language problems had additional medical issues. A majority of 67.06% agreed that excessive screen time may cause language delay in children, while 14.46% disagreed, and 18.48% were neutral. Notably, 51.42% agreed that early intervention can lower the cost and duration of treatment for delays in speech and language. Parents who had good knowledge regarding speech-language delay (≥7 correct answers) represent 9% of the study population. The overall knowledge score was 5.77 ± 2.40. Figure 1 represents parents' knowledge as an overall knowledge of language milestones and speech-language delay.

**Table 2:** Knowledge Assessment on Language Milestones and Speech-Language Delay in Children All values are in n(%) unless otherwise indicated.

Statements/Questions			Responses				Values	
									Knowledge Regarding Language Milestones								
By what age should a child be able to vocalize (cooing)?			1 month				73(17.30)										
			4 months				212(50.24)	
			8 months				69(16.35)	
			12 months				15(3.55)	
			I don't know				53(12.56)	
By what age does a child say “Mama” or “Baba”, nonspecific?			9 months				254(60.19)	
			12 months				108(25.59)	
			18 months				30(7.11)	
			24 months				6(1.42)	
			I don't know				24(5.69)	
By what age should a child be able to call his mother or father “Mama” and “Baba” respectively?			9 months				63(14.93)	
			12 months				146(34.60)	
			18 months				137(32.46)	
			24 months				45(10.66)	
			I don't know				31(7.35)	
By what age should a child be able to follow a one-step verbal command, such as "sit down" or "give me the toy"?			6-9 months				45(10.66)	
			12-15 months				201(47.63)	
			18-24 months				114(27.01)	
			3 years				39(9.24)	
			I don't know				23(5.45)	
By what age should a child have a vocabulary of 50 or more words?			12-18 months				49(11.61)	
			24-30 months				130(30.81)	
			3 years				133(31.52)	
			4 years				61(14.45)	
			I don't know				49(11.61)	
General Knowledge About Speech and Language Delay								
									Have you ever heard of speech and language delay in children?			No				35(8.29)	
Yes				387(91.71)													
						How would you describe your understanding of speech and language delay?						Basic				166(42.89)	
Moderate				187(48.32)													
									Advanced				34(8.79)	
Which of the following best describes expressive language delay?			Difficulty with understanding spoken language												41(9.72)	
									Difficulty with producing speech sounds				92(21.80)			
			Difficulty with forming sentences and expressing ideas												214(50.71)	
							Difficulty with following directions				14(3.32)					
			I don't know										61(14.45)	
							At what age do most children point to ask for something or to get help?			6 months					94(22.27)	
			15 months							177(41.94)	
			18 months							74(17.54)	
			30 months							28(6.64)	
			I don't know							48(11.61)	
Receptive language involves which of the following?			The ability to understand and comprehend spoken or written language				191(45.26)	
									The ability to express oneself verbally or in writing				25(5.92)	
			The ability to use appropriate body language and gestures				82(19.43)							
									The ability to initiate and maintain conversations				22(5.21)	
			I don't know				102(24.17)							
									At what age do children typically start looking when their name is called?			3 months				88(20.85)	
			9 months				180(42.65)				
			12 months				82(19.43)				
			18 months				30(7.11)				
			I don't know				42(9.95)				
Speech and language milestones develop at a slower rate in males compared to females.			Agree				146(34.60)	
			Strongly agree				41(9.72)	
			Disagree				59(13.98)	
			Strongly disagree				22(5.21)	
			Neutral				154(36.49)	
Spending too much time in front of screens may lead to language delay in children.			Agree				180(42.65)	
			Strongly agree				103(24.41)	
			Disagree				50(11.85)	
			Strongly disagree				11(2.61)	
			Neutral				78(18.48)	
Breastfeeding protects against delays in young children’s language development.			Agree				150(35.55)	
			Strongly agree				52(12.32)	
			Disagree				61(14.45)	
			Strongly disagree				5(1.18)	
			Neutral				154(36.49)	
Most children with language delays have other associated medical findings.			Agree				159(37.68)	
			Strongly agree				19(4.50)	
			Disagree				83(19.67)	
			Strongly disagree				12(2.84)	
			Neutral				149(35.31)	
Some children with speech and language delays will catch up and display more typical language skills as they age without any interventions.			Agree				162(38.39)	
			Strongly agree				13(3.08)	
			Disagree				114(27.01)	
			Strongly disagree				14(3.32)	
			Neutral				119(28.20)	
Early intervention can decrease the cost and duration of treatment of speech and language delay in children.			Agree				217(51.42)	
			Strongly agree				142(33.65)	
			Disagree				8(1.90)	
			Strongly disagree				2(0.47)	
			Neutral				53(12.56)	
Overall knowledge score for language milestones and speech and language delay (mean ± SD).							5.77 ± 2.40	

**Figure 2:**
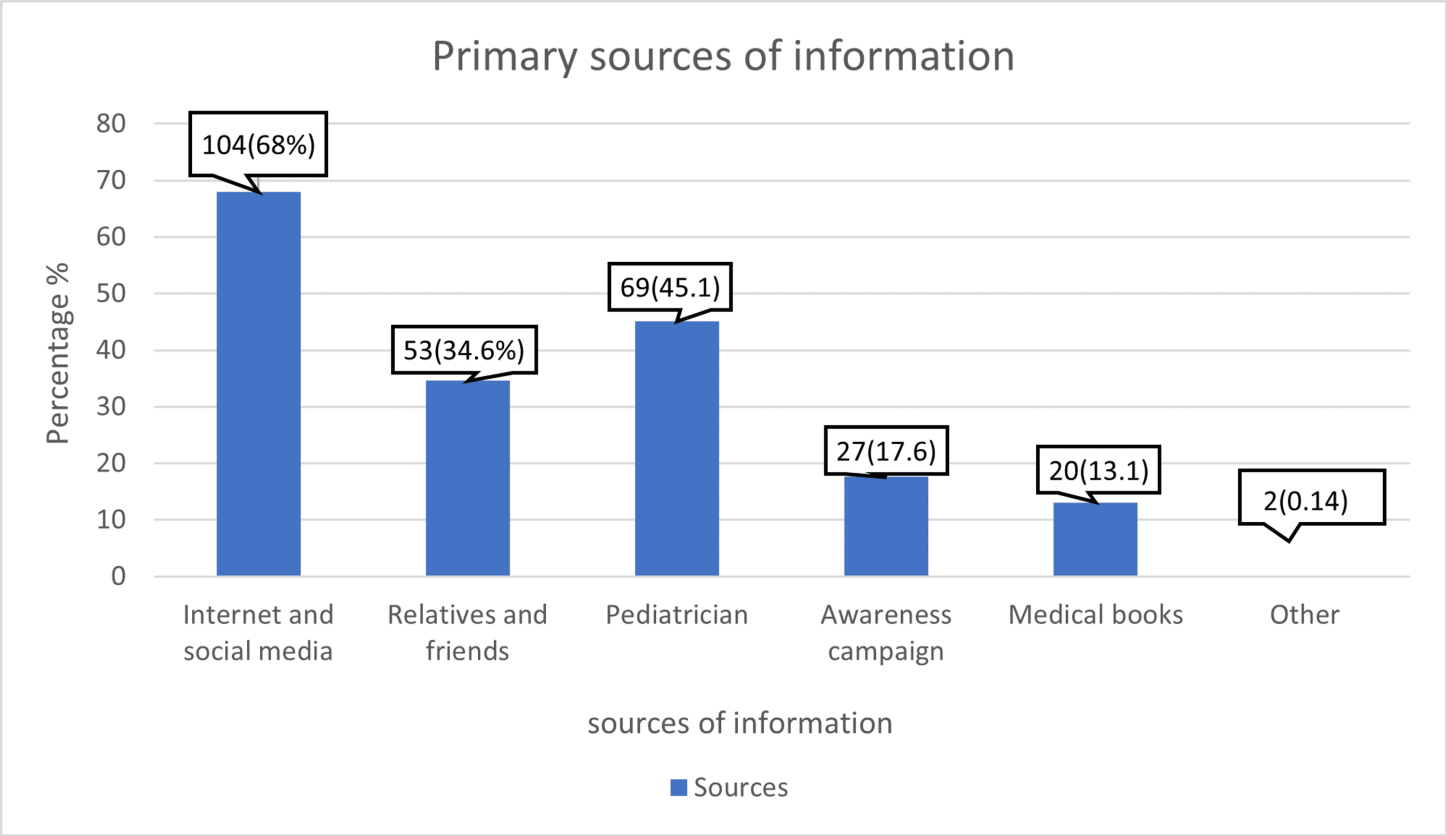
Primary Sources of Knowledge About Speech and Language Delay Among Participants

Table 3 shows that most participants (62.56%) never searched for information about children’s language milestones. However, the primary sources for obtaining information were the internet and social media (68%), followed by pediatricians (45.1%). Figure 2 summarizes parents' sources to obtain knowledge regarding speech and language delays in their children. Regarding pediatrician visits for checkups and vaccinations in the child’s first two years, 29.15% attended more than nine appointments. Additionally, 32.23% of parents spent more than eight hours per day interacting with their children to improve developmental milestones. When observing potential speech and language difficulties, only 21.80% would seek help within one month.

**Table 3:** Parental Practices Related to Language Milestones and Speech-Language Delay

Statements					Responses			N(%)	
										Have you personally searched for information about children’s language milestones?					Yes			158(37.44)	
No			264(62.56)																
					If yes, what is your primary source for obtaining information concerning children’s language milestones?										Internet and social media			104(68)	
Relatives and friends			53(34.6)																
										Pediatrician			69(45.1)	
Awareness campaign			27(17.6)											
										Medical books			20(13.1)	
Other			2(0.14)											
										How many times did you visit the pediatrician during your child's first two years for routine check-ups such as examination and vaccination?					Less than 9 visits			105(24.88)	
9 visits			27(6.40)																
															More than 9 visits			123(29.15	
Not sure			167(39.57)																
															How much time do you spend with your child daily?					Less than 1 hour			13(3.08)	
1 to 2 hours			60(14.22)																					
					2 to 4 hours			105(24.88)											
4 to 8 hours			108(25.59)																
					More than 8 hours			136(32.23)											
How soon would you take action or seek professional help after first observing symptoms of a potential speech-language delay in your child?										Within 1 month			92(21.80)						
					Within 3 months			92(21.80)											
										Within 6 months			83(19.67)						
					Within a year			80(18.96)											
										More than 1 year			75(17.77)						

Table 4 reveals a significant relationship between knowledge of children’s language milestones and speech and language delay with two key factors. Participants with better knowledge were more likely to visit a pediatrician more than nine times in their child's first two years (χ² = 12.7, p = 0.03). Additionally, those with sufficient knowledge were more likely to seek professional help within one month of observing indications of speech-language delay (χ² = 17.26, p = 0.02). There was no statistically significant association between time spent with the child and knowledge level (χ² = 7.83, p = 0.2).

**Table 4:** Association Between Knowledge Levels and Parental Practices * Statistical significance (p<0.05) χ² chi-square value

Statement			Knowledge categories			χ²	P-value
			Poor	Acceptable	Good		
Have you personally searched for information about children’s language milestones?		Yes	86 (54.43%)	69 (43.67%)	3 (1.90%)	4.14	0.1
		No	168 (63.64%)	94 (35.61%)	2 (0.76%)		
How many times did you visit the pediatrician during your child's first two years for routine check-ups such as examination and vaccination?		< 9	69 (65.71%)	36 (34.29%)	0	12.7	0.03*
		9 Visits	10 (37.04%)	17 (62.96%)	0		
		> 9	68 (55.28%)	52 (42.28%)	3 (2.44%)		
		Not Sure	107 (64.07%)	58 (34.73%)	2 (1.20%)		
How much time do you spend with your child daily?		> 1 Hour	10 (76.92%)	3 (23.08%)	0	7.83	0.2
		1-2 Hours	43 (71.67%)	17 (28.33%)	0		
		2-4 Hours	63 (60%)	40 (38.10%)	2 (1.90%)		
		4-8 Hours	67 (62.04%)	40 (37.04%)	1 (0.93%)		
		> 8 Hours	71 (52.21%)	63 (46.32%)	2 (1.47%)		
How soon would you take action or seek professional help after first observing symptoms of a potential speech-language delay in your child?		1 Month	47 (51.09%)	44 (47.83%)	1 (1.09%)	17.26	0.02*
		3 Months	45 (48.91%)	46 (50%)	1 (1.09%)		
		6 Months	58 (69.88%)	23 (27.71%)	2 (2.41%)		
		1 Year	55 (68.75%)	24 (30%)	1 (1.25%)		
		> 1 Year	49 (65.33%)	26 (34.67%)	0		

## Discussion

The aim of this study was to assess parents' awareness, knowledge, and practices regarding speech and language delay in children in the Eastern Region of Saudi Arabia. The findings indicate a good awareness of the term “speech and language delay” with significant gaps in specific knowledge areas. The 16-question questionnaire evaluating parents' overall knowledge of language milestones and speech-language delay showed that 60.19% of parents have poor knowledge, 38.63% have acceptable knowledge, and only 1.18% have good knowledge.

Many studies have been conducted in various countries, including Pakistan, Saudi Arabia, Iraq, and Egypt, to assess parents' knowledge of language milestones. In some studies, knowledge of language milestones ranged from 60% to 74.49%, which is considered acceptable. For instance, a study by Alkhazrajy and Aldeen (2017) in Iraq assessed mothers’ awareness of developmental milestones in children under two years and found that knowledge was highest (59%) for language and hearing developmental milestones [15]. Another study conducted in Al-Kharj, Saudi Arabia, by Elsayed et al. (2023) evaluated parents' knowledge of developmental milestones, showing that parents had an overall knowledge of 60% regarding language development [16]. In Sohag City, Egypt, a study by Mostafa and Ahmed (2018) evaluated public awareness and attitudes toward delayed language development. In their study, out of 1,380 respondents, 74.49% were found to have good knowledge [17].

Other studies have reported inadequate knowledge of language milestones, similar to our findings. For example, Kumar et al. (2024) found that 42% of parents in Pakistan displayed insufficient knowledge of language milestones [18]. Additionally, Alzarani et al. (2024) demonstrated that 26.7% of parents had good knowledge of developmental milestones, while 26.5% had only fair knowledge. They also reported that 46.60% of parents in Saudi Arabia had poor knowledge of developmental domains, including language [19]. A study by Alghamdi et al. (2023) revealed that parents had lower levels of knowledge in language and social domains compared to motor and cognitive domains [14].

The variation in knowledge reported in the literature can be attributed to the different study designs. The difference between the current study and others is focusing on either language milestones or delayed language, while the current study assesses parents' knowledge in a questionnaire that contains both aspects. Also, these studies differ from each other according to the number of questions and their scoring system for assessing language milestones or for assessing delayed language development, which gives us different results and different labeling of knowledge for each study's result. The variation in awareness can also be attributed to cultural and contextual differences.

In the current study, the primary sources for obtaining information were the Internet and social media (68%), followed by pediatricians (45.1%). According to Alghamdi et al. (2023), the majority of parents (54%) relied on online resources and their general practitioners/pediatricians (51%) for information about their children's developmental milestones [14]. Also, according to Elsayed et al. (2023), most parents would rely on parents and friends (38.9%), followed by media and social networking (31.5%) [16]. This information indicates that parents often struggle to identify developmental delays, including speech delays accurately. This is consistent across regions due to limited access to early childhood developmental information and professional guidance, as most of them seek information from social media and their families, as is shown in our study and others.

By looking into the studies mentioned regarding speech and language awareness, parents know that intervention at any sign of delay would benefit their children. The current study demonstrates that 51.42% of parents agreed that early intervention could lower the cost and duration of therapy. Similarly, 55.1% of parents in Pakistan said they would see a pediatrician if they observed signs of speech delay [18]. Also, according to Alzahrani et al. (2024), most participants are willing to undergo screenings if risk factors exist [19]. The current study showed that only 21.80% of parents in Saudi Arabia said they would take action within one month when a symptom is observed. The reason for that low percentage could be that parents believe some children with speech and language delays will catch up and display more typical language skills as they age without any interventions.

A lot of research has examined screen time and language development, with mixed results on whether it causes delays or might help in some cases [20-22]. According to the current study, 67.06% of parents responded positively that screen time might lead to language delays. This could be due to personal observations of their children's development, as parents frequently notice that excessive screen use affects the interaction between them and their children.

The current study highlighted an important relationship between knowledge and practice. The study found a relationship between parents' knowledge of language milestones and speech-language delay with the number of pediatrician visits and how soon they would take action if they noticed any potential signs of language delay.

This cross-sectional study provides us with a better understanding of parents’ awareness and knowledge levels regarding speech and language delays in children, which are essential for addressing public health needs in this area. Healthcare facilities and community organizations can create more effective strategies to improve parents’ knowledge about speech and language delays in children by identifying gaps in parental understanding. This study also highlights parents’ actions in seeking help after first observing symptoms of a potential speech and language delay in their children, as only 21.80% of parents seek professional help within one month. This finding shows that parents do not have enough knowledge about early intervention for speech and language delays. Creating effective strategies can be achieved by teaching medical professionals to have conversations with parents about the early detection of speech and language problems.

Limitations

Regarding the limitations of this study, the majority of participants were women. It is reasonable to argue that women are generally more likely to participate in surveys and that they have a greater interest in subjects involving the growth and development of children. The Eastern Region had a higher number of participants than other regions, which notably had smaller numbers. A justification for this could be that more participants may have been recruited in the Eastern Region due to the researchers' Eastern background.

In the current study, parental knowledge of speech delays is insufficient, and early intervention is recognized as key. More targeted educational campaigns and pediatric partnerships are crucial to addressing these knowledge gaps and improving early detection of speech delays.

This analysis offers a strong base for discussing the importance of increasing parental knowledge, addressing misconceptions, and improving healthcare communication in the research. Further research is needed to evaluate the impact of educational programs or awareness campaigns on improving parents' knowledge and early identification of speech and language delays. It should also investigate factors that influence awareness and knowledge in depth, such as measuring the socioeconomic status and educational level across the regions of Saudi Arabia. Further research should assess the knowledge and practices of pediatricians, family physicians, and other healthcare providers in identifying and managing speech and language delays. Additionally, study the long-term outcomes of children who receive early intervention for speech and language delays compared to those who do not. Also, future research should conduct clinical validation to measure if knowledge and practices align with actual speech-language development in their children.

## Conclusions

The result of our study showed that most parents have poor knowledge regarding language milestones and speech and language delay. We found a significant relationship between parents' knowledge and their practice; as parents' knowledge improved, they visited the pediatrician more frequently and had a faster response time to signs of speech delay. It can be hard for parents to detect any signs of delay, which may be due to the sources of information on which parents depend. Current studies, including ours, reported that most parents rely on social media and their relatives and friends, which affects their practices with their children. The importance of our study is that it assesses not only knowledge of language milestones but also emphasizes parents' understanding of speech-language delays and how they will act accordingly. This study contributes to expanding research on this topic, helping us to predict parents' actions and what interventions must be undertaken by healthcare providers. It also creates opportunities for other studies to be conducted to ensure the best outcomes for children's language development.
